# Dr. Turner's Elements of Chemistry

**Published:** 1847-04

**Authors:** 


ART. V.—*Elements of Chemistry, including the Actual State and Prevalent Doctrines of the Science.* By the late EDWARD TURNER, M.D. F.R.S. L. & E. Eighth Edition. Edited by Baron LIEBIG, Professor of Chemistry in the University of Giessen, and WILLIAM GREGORY, M.D. F.R.S.E., Professor of Chemistry in the University of Edinburgh. Part I. *Inorganic Chemistry.—London,* 1847, 8vo. pp. 676.

THAT this work should so completely hold its ground, in spite of the competition of such Treatises as those of Professors Graham and Kane, to say nothing of the excellent Manuals of Fownes and Gregory, is of itself a sufficient testimony of the excellence of its original plan, and of the completeness with which it has been kept by its present editors, *au courant* with the rapid progress of Chemical Science. It is scarcely requisite, therefore, that we should do more than notice the appearance of the first part of a new edition; in the preparation of which Professor Gregory has obviously been at great pains to embody every new discovery of sufficient note, relative to the departments which it includes. The remaining portion of the work, embracing organic chemistry, is announced as speedily forthcoming.

The following extract from the Preface will be interesting to many of our readers, as affording a marked proof of that growing conformity of opinion upon fundamental questions, which is one of the most satisfactory indications of the real progress of science:

"Since the publication of the last edition, many continental chemists, including Liebig, Wöhler, Gmelin, and their numerous pupils, have finally adopted the British equivalents or atomic weights for those substances in regard to which a difference existed. This difference, therefore, no longer exists as far as concerns the chemists above named, and many others; who in their works now admit as we do water to be H O, not as formerly $H_2$ O; and chloride of potassium to be K Cl, and not as formerly K $Cl_2$, &c. In short, the atomic weights of hydrogen, nitrogen, chlorine, bromine, iodine, and fluorine are now, by those chemists, assumed to correspond with their equivalents, where formerly the equivalent was made to contain two atoms." (p. iii.)

On the other hand, the editors have abandoned the old mode of regarding the combining equivalents of phosphorus, arsenic, and antimony, as twice their atomic proportion; and have thus substituted the simple formulæ $PO_5$, As $O_3$, and Sb $O_3$ for the more complex $T_2$ $O_5$, $As_2$ $O_3$, and $Sb_2$ $O_3$, hitherto employed.

